# Supplementary figures and images for: Interaction hub critical for telomerase recruitment and primer-template handling for catalysis
Source: Life Sci Alliance. 2023 Mar 24;6(6):e202201727. doi: 10.26508/lsa.202201727 (PMC10055720; doi:10.26508/lsa.202201727)

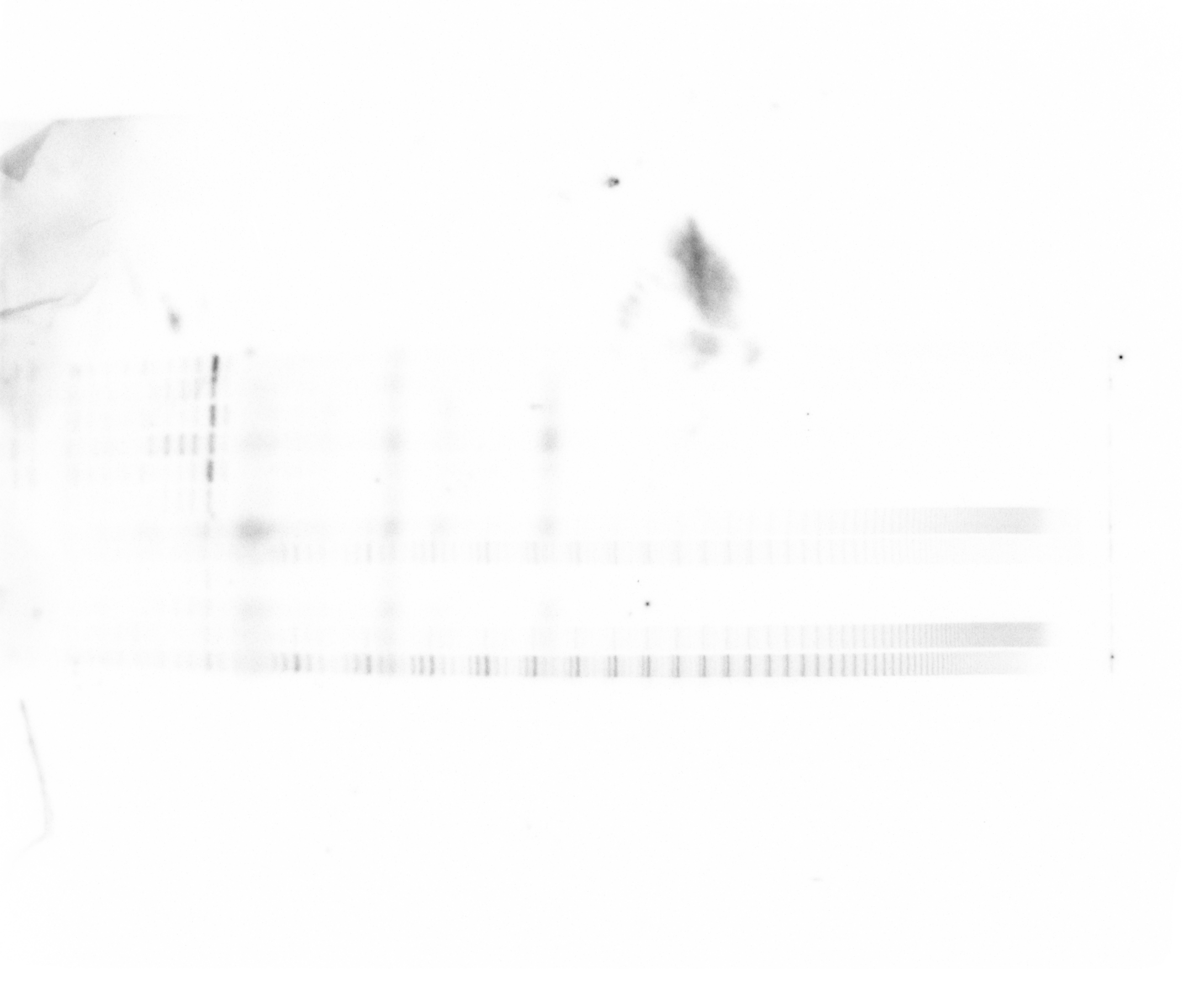

Supplement: Supplementary file 1 [file LSA-2022-01727_SdataF1.tif]

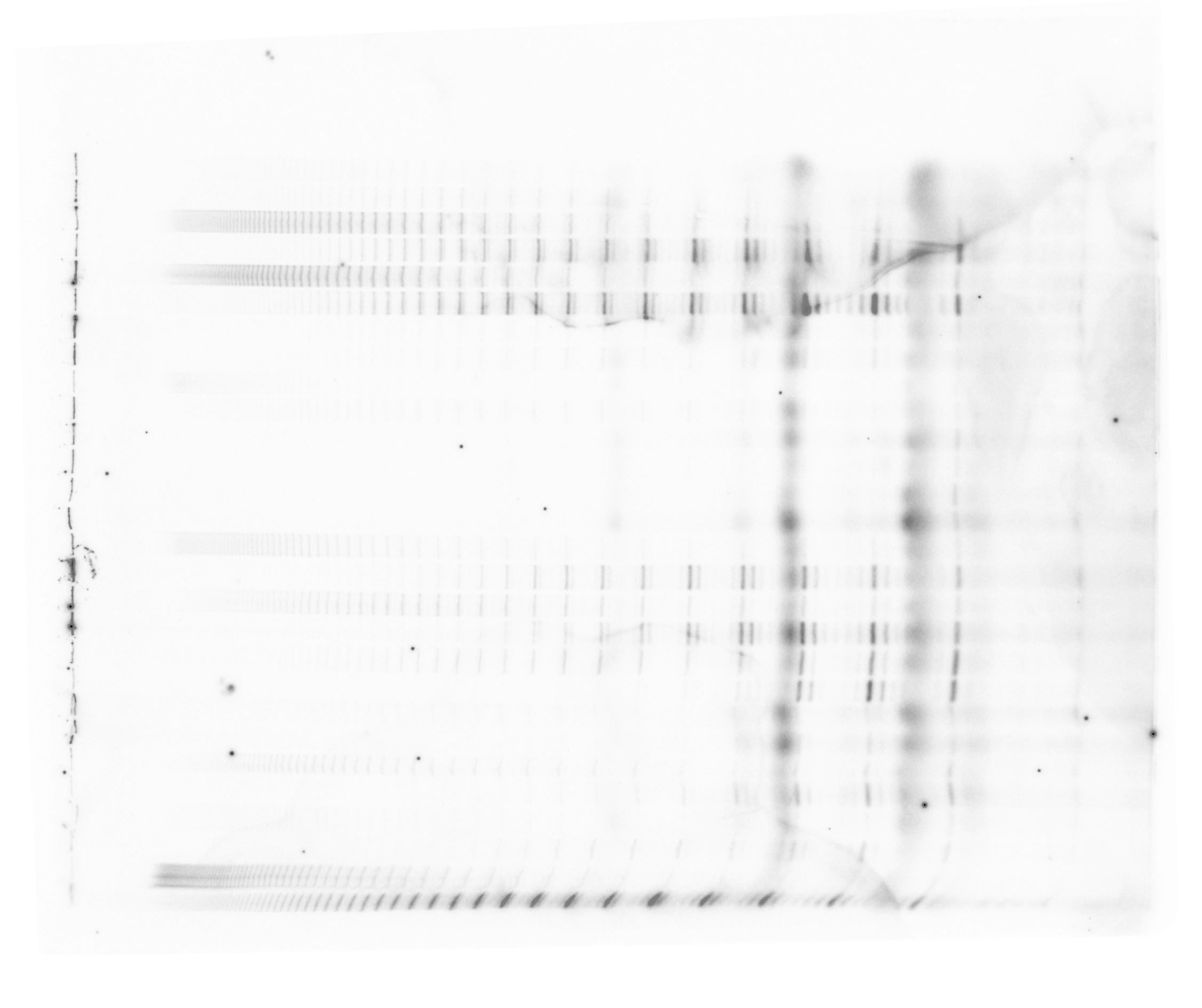

Supplement: Supplementary file 4 [file LSA-2022-01727_SdataF3.2.tif]

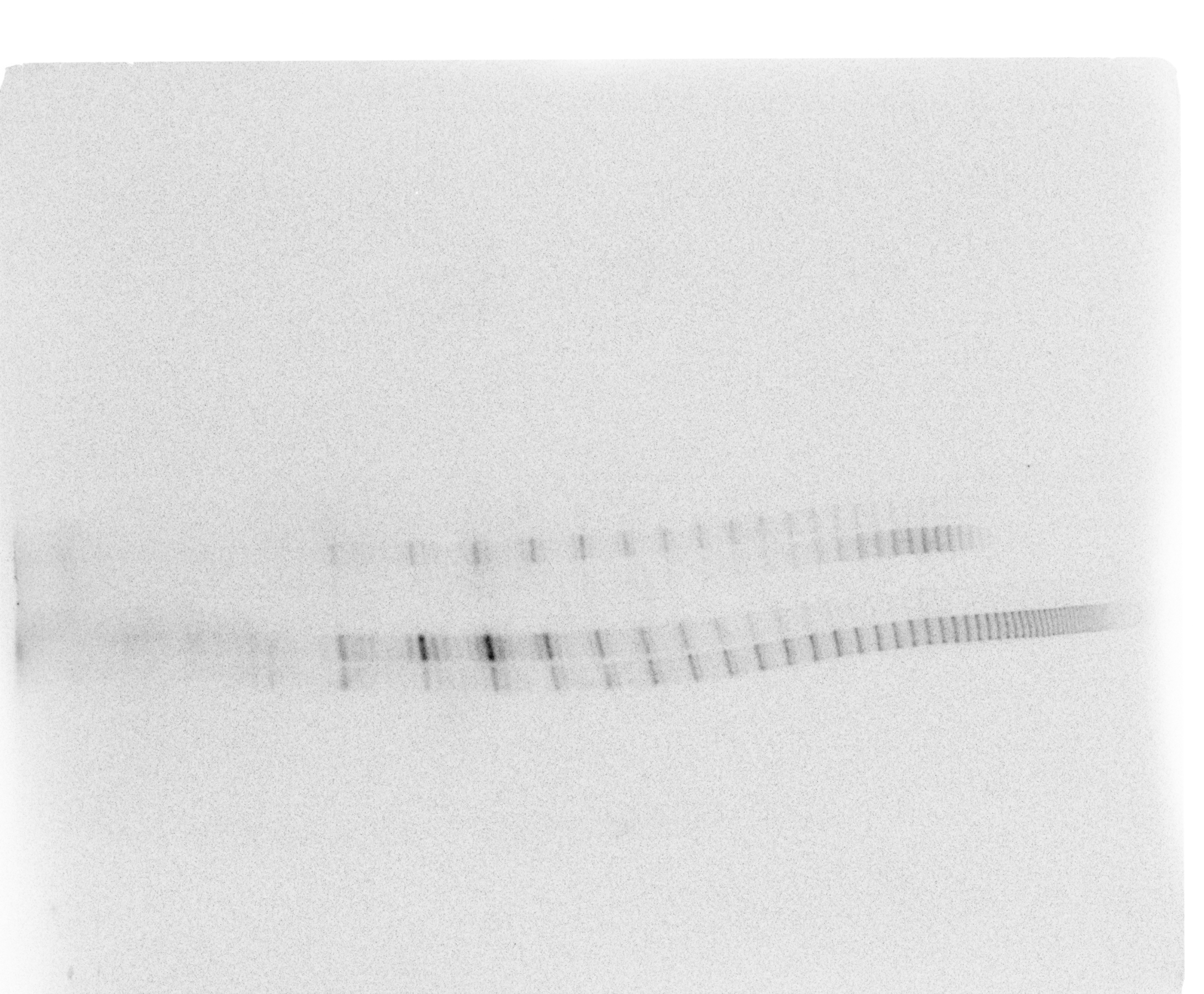

Supplement: Supplementary file 5 [file LSA-2022-01727_SdataF3.3.tif]

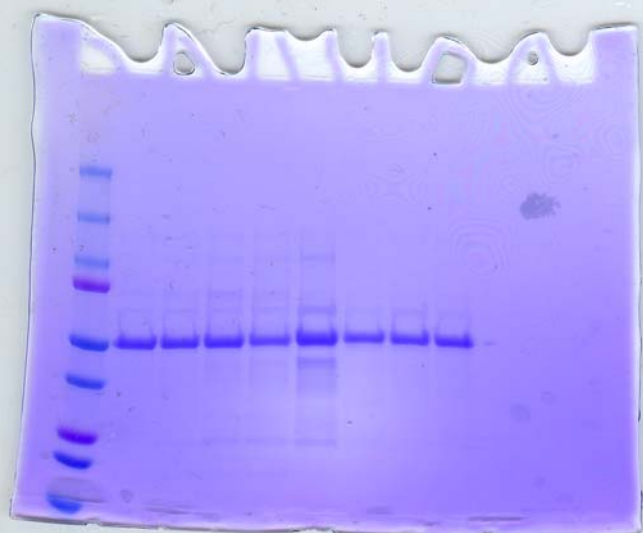

Supplement: Supplementary file 9 [file LSA-2022-01727_SdataF5.2.pdf]

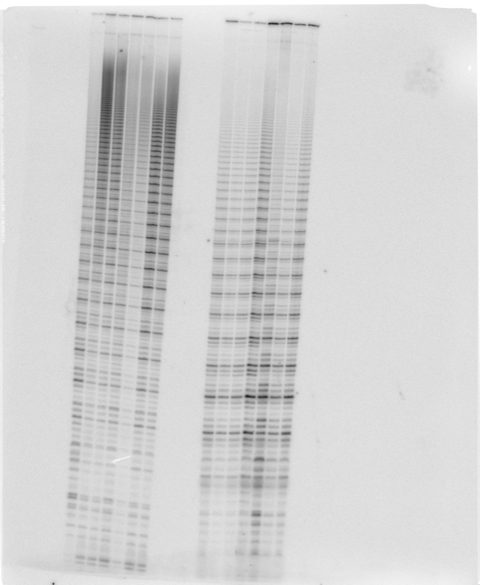

Supplement: Supplementary file 10 [file LSA-2022-01727_SdataF5.3.tif]
