# Supplementary material for: Interaction hub critical for telomerase recruitment and primer-template handling for catalysis
Source: Life Sci Alliance. 2023 Mar 24;6(6):e202201727. doi: 10.26508/lsa.202201727 (PMC10055720; doi:10.26508/lsa.202201727)
Supplement: Supplementary file 2 [file LSA-2022-01727_SdataF2.pdf]

Fig 2B transcomplementation IF quantification

TERT	TERTΔTEN	TERTΔTEN + TEN	TERTΔIFD	TERTΔTENΔIFD	TERTΔTENΔIFD + TEN
**91.8556444**	2.56410256	43.5897436	0	12.8205128	14.7435897
**94.6153846**	0	19.2307692	0	2.56410256	13.4615385
**98.9010989**	0	43.3333333	0	0	0
