# Supplementary material for: Interaction hub critical for telomerase recruitment and primer-template handling for catalysis
Source: Life Sci Alliance. 2023 Mar 24;6(6):e202201727. doi: 10.26508/lsa.202201727 (PMC10055720; doi:10.26508/lsa.202201727)
Supplement: Supplementary file 3 [file LSA-2022-01727_SdataF3.1.pdf]

Fig 3B IFD mutation panel processivity quantication + PT lanes

WT	K749E	R756E	K757E	F759E	K760E	H762E	F776E	L786E	D788R
**0.50879**	0.369453	0.320793	0.203158	0.199718	0.269501	0.499718	0.229079	0.239126	0.283557
**0.554671**	0.325139	0.34624	0.179349	0.094687	0.301299	0.458955	0.146742	0.1406	0.46741
**0.39286**	0.142375	0.195215	0.207296	0.056956	0.220829	0.287844	-0.33789	0.133442	0.186042

Fig 3E Motif 3N normalized activity

WT	WT+PT	Δ641-651	Δ641-651+PT
**1**	1.108778	2.794098	1.207795
**1**	1.062298	1.365823	1.979337
**1**	1.020584	2.101151	2.765274

Fig 3C IFD mutation panel relative activity quantification - PT lanes

WT	K749E	R756E	K757E	F759E	K760E	H762E	F776E	L786	D788R
1	1.132561	1.004672	0.674734	0.695901	0.539852	1.387196	0.976458	0.573581	0.606591
1	0.147964	0.123393	0.105502	0.128053	0.274883	0.220485	0.11072	0.042355	0.065442
1	0.420697	0.270876	0.501341	0.335026	0.182804	0.432991	0.168121	0.128089	0.031861
