# Supplementary material for: Interaction hub critical for telomerase recruitment and primer-template handling for catalysis
Source: Life Sci Alliance. 2023 Mar 24;6(6):e202201727. doi: 10.26508/lsa.202201727 (PMC10055720; doi:10.26508/lsa.202201727)
Supplement: Supplementary file 6 [file LSA-2022-01727_SdataF4.1.pdf]

Fig 4B F99V F101V processivity quantification

WT	+ obdbd	wt + pt	f99v	f99v+obdbd	f99v+pt	f101v	f101v +obdbd	f101v+pt
**0.0814672**	0.465928996	0.468414246	0.065481893	0.162854521	0.276302384	0.044639217	0.392592549	0.448652258
**0.227108657**	0.639471779	0.771674562	0.234172067	0.574929391	0.578012383	0.316751444	0.564633527	0.671669424
**0.31535124**	0.815317463	0.729849236	0.269192298	0.61281108	0.355323208	0.290803526	0.665810614	0.435222288

Fig 4E F99V IF

WT	F99V
**96.386306**	0
**100**	0
**98.7179487**	0

Fig 4F F101V IF

WT	F101V
**97.7350031**	25
**97.7678571**	26.9875957
**99.2857143**	18.9285714
